# Laser light absorption of high-temperature metal surfaces

**DOI:** 10.1016/j.heliyon.2023.e21021

**Published:** 2023-10-16

**Authors:** Joerg Volpp

**Affiliations:** Department of Engineering Sciences and Mathematics, Luleå University of Technology, 97187, Luleå, Sweden

**Keywords:** Laser treatment, Molecular dynamics, Laser light absorption

## Abstract

Laser beam absorption is the basic effect to enable many high-temperature applications and processes. However, high temperature absorption data of metals is often not available or based on theoretical assumptions. In this work, using a newly developed experimental arrangement to measure laser light absorption on liquid metal surfaces even above boiling temperature enabled the derivation of absorption values in those regimes. Results indicate that interband absorption must be considered even at such high temperatures against common theoretical predictions. It is shown that the simulated nearly constant absorption depth and absorption values between melting and boiling temperatures indicate that the increased atom distance due to thermal expansion, denoting a reduced absorption volume, is counterbalanced by the increased statistical availability of conduction electrons due to Fermi band broadening.

## 1 Introduction

Absorption of laser light is used for many industrial processes. Typical applications can be found in laser welding, cutting, marking, Additive Manufacturing and more. For understanding material behavior in general and making reliable predictions in simulations of the process behavior, an elementary part is to know how much energy is actually transferred into the material. In particular for metals, the absorption behavior is difficult to theoretically predict due to the complex atomic structures and difficulties to measure absorption at high temperatures. Therefore, absorption is typically measured on solids and extrapolated to higher temperatures using assumptions. However, there are multiple modeling approaches that estimate the absorption during laser processing at high temperatures. E.g. Abadi et al. [1] developed a model predicting the absorptance based on surface conditions and temperature, finding that typical assumptions underestimate the absorption. Similarly, Ebrahimi et al. [2] observed an increase in absorption values of 316L stainless steel at increasing temperatures below boiling temperature. Those measurements give a good insight into the overall absorptance of a certain laser process including all absorption effects such as impacts of surface roughness or multiple reflections, but shows challenges to determine and relate temperature data to the direct surface absorption.

Typical methods to measure metal absorption include laser calorimetry, integrating spheres or emittance spectroscopy [[3], [4], [5]]. Theoretical approaches are usually based on descriptions of bonding characteristics solved with Embedded Atom (e.g. Refs. [6,7]) or Molecular dynamic simulations (e.g. Refs. [8,9]). This work aims to provide additional information about absorption characteristics of steel at high temperatures.

Literature shows results of experimental and theoretical works on laser light absorption at different impacting factors, such as different laser light wavelengths, different surface roughness and partly elevated temperatures. Two main absorption measurement techniques are usually distinguished, namely direct calorimetry measurements and indirect radiometric absorption measurements. Calorimetric methods use the measurement of a temperature increase due to light illumination. E.g. Fuerschbach & Eisler [10] used calorimetric measurements of 304 stainless steel and detected variations of the absorption from 0.38 to 0.67. Trapp et al. [11] used micro-calorimetry in order to measure laser light absorption of high-temperature metal surfaces, focusing on powder material during laser powder bed fusion processing and found the general trend of a decreased absorption when the powder is melted and an increased absorption when a keyhole is formed at high laser intensities. This was valid for aluminium, steel and titanium [12].

Calorimetry uses thermocouples (e.g. Ref. [13]) or evaporation characteristics, e.g. radiation from helium vaporization by Daunt & Keeley [14]. However, thermocouple measurements require a sufficiently accurate model considering heat conduction and dissipation [15]. Introducing water calorimeters, the measurement accuracy could be increased [16].

**Fig. 1:**
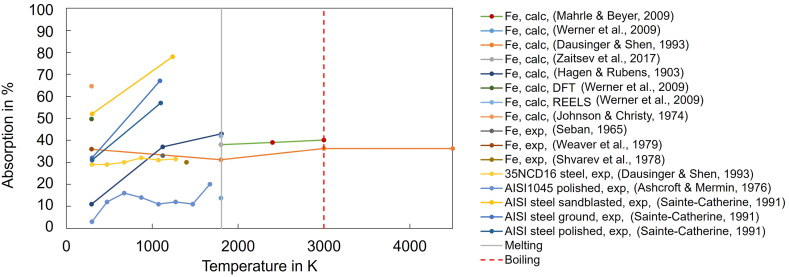
Collection of experimental absorption measurements (exp) and theoretical calculations (calc) from literature at elevated temperatures of iron (Fe) and steel at different surface conditions (perpendicular laser incidence).

Since the absorption A is connected to the reflectivity R of materials by A=1−R, (neglecting transmission effects, which is typically valid for metals), reflectivity measurements can give information about the absorptivity (Fig. 1). With an integrating sphere, the spectral reflectivity can be derived [17], although high-temperature surfaces are challenging to measure. However, spectral reflectivity measurement was shown to be feasible in the range between 2000 K and 4000 K using an integrating sphere [18] with monochromatic light, while the absorptivity showed a slight increase with increasing wavelength and a distinct increase with increasing temperature above the melting point [19]. Adapted reflectometry measurements were developed using the change of polarization of laser light when reflected from a sample surface [20]. This ellipsometer method was able to determine temperature dependent absorption values of several materials also slightly above melting temperatures. Combined absorption measurements using an integrating sphere with in-situ X-ray observation of the vapor capillary revealed the impact of different keyhole geometries on the light absorption [21]. The dynamic absorptance during laser material heating of 316L stainless steel measured with a high-resolution integrating sphere gave the absorption values when changing the welding mode from conduction to deep penetration [22]. The comparison with data derived from calorimetric measurements indicated an underestimation of absorption values in the calorimetric data.

Early theoretical approaches such as the Drude model [23], Hagen-Rubens [24] or Bramson theory [25] predict the absorptivity in solid state for far infrared wavelengths. The reflectivity was shown to decrease with increasing temperature (e.g. Ref. [26]).

The absorption theory of Drude's early work was derived from the elementary electron theory of metals. It was assumed that only electrons in the conduction band absorb the photonic energy and are accelerated by the induced electric field of the light, while collisions with lattice imperfections damp the electrons again. Since this damping effect is also the origin of electrical resistivity, the relation between conductivity and absorptivity was explained by Dausinger & Shen [27]. Drude's theory can describe the absorption of many metals in the infrared wavelength but shows deviations in the visible and ultraviolet range [28]. However, it was not possible to fit all metals, like iron [27]. More complex models were developed considering e.g. powder surfaces and multiple reflections in order to get a better understanding of the impacts of real surfaces on the total absorption [29]. Multi-physics absorption calculations in complex dynamic keyholes were conducted showing that smaller keyholes show increased absorption due to increased multiple reflections [30]. However, often the parameter absorption needs to be used as model parameter to adapt the results to the experimental validation data.

Further, it was found that the optical coefficients n (refraction index) and k (extinction coefficient) that describe the optical characteristics of materials and their interfaces are related to the electronic structure of solids [27]. Therefore, the absorption coefficient can be derived when knowing the complex refractive indexes using Fresnel's equations or directly solving the Helmholtz equation [31]. However, the theory works well only for several materials at low temperatures [20]. Unfortunately, the refractive index is unknown for most materials at and above melting temperatures [32] and even the temperature dependent Drude model cannot sufficiently describe the absorption at high temperatures [20].

In this work, it is aimed to increase the knowledge about absorption values of metal surfaces at high temperatures focusing on direct absorption values without extensive melt pool movement, Marangoni effect impacts, complex powder surfaces or deformed surfaces as basis for fundamental absorption calculations for improved energy transfer descriptions. A direct relation of absorption values to temperatures is aimed for.

## 2 Methods

### 2.1 Reflectivity measurements

In order to derive absorption values of high-temperature metal surfaces, reflectivity measurements were conducted [33]. The setup consisted of a heating laser beam and a measuring laser beam. The heating and melting of the material were achieved using fibre laser (IPG YLR-15000, wavelength 1070 nm at 1.5 kW output power). The material used was the low carbon steel S275 (0.15 % C, <1.5 % Mn, <0.5 Cu) with milled surface. The measurement beam (Cavilux, cw, 808 nm wavelength, multimode, no preferred polarization, spot diameter on material: ∼10 mm) was positioned at an angle onto the molten material to be reflected into the intensity sensor (RedLake Mono N4–S2, recording at 500 fps). A notch-filter ensured that only the reflected light from the measuring beam was reflected into the sensor. The recorded grey-scale values were related to reflected intensity values. The uncertainties of the measured values were estimated considering light scattering losses (3 % based on geometrical estimations of the rays missing the melt pool due to its slight curvature), pixel intensity resolution (∼4 %) and setup uncertainties due to the alignment of the measuring beam with the intensity sensor (∼1 %). Those uncertainties were added to the value variations considering the minimum and maximum values.

A thermal sensor (DFK 23GM021 by TheImagingSource) was installed to measure the surface temperature on the melt pools. Two channel data was evaluated derive data independent from emissivity [33]. Temperature measurement at high temperatures is difficult and incorporates uncertainties. Depending on the measured temperature value, the uncertainty of the measured absolute temperature is between 1.1 % and 25 % [33].

In parallel, high-speed imaging was conducted in order to observe the melt pool surface and dynamics. For that, a high-speed-imaging camera (Fastcam Mini UX100 type 800 K-M-16G at 12 500 fps) was used. Measurements, recorded when the melt pool surface oscillated, were excluded from the analysis. Further uncertainties are related to the scattering of light from the material surface and the temperature measurement. The suggested method is able to detect the directed reflections from the melt pool surface. Scattered light from roughness of the surface can decrease the total reflected light to the sensor. However, in the liquid state, the molten surfaces were macroscopically smooth and when oscillations occurred, the recorded values were excluded from the analysis.

To protect the melt pool material, Argon shielding gas was applied at 5 L/min perpendicular to the material surface based on best practise experience and previous calculations of gas flows during laser beam welding to enable a coverage of the processing zone and avoid extensive surface oxidation, which can alter the absorption values. It cannot be completely excluded that ambient gas is temporarily transferred to the processing zone due to the dynamic process. However, the high-speed imaging videos show no visible oxidation during the time of measurements.

### 2.2 Molecular dynamic simulation

A simplified molecular dynamic model was used to simulate the atom positions of the lattice. Pure iron without contamination was assumed arranging an initial ordering of 10 × 10 × 10 atoms in a cube with constant initial distance. A Lennard-Jones potential (e.g. Refs. [8,34]) was applied, including additional consideration of long-range retractive Coulomb forces between the atoms to consider the complex metallic structure [35]. The starting points of the 10 × 10 × 10 atoms lattice is the arrangement at atom distances based on the actual temperature for the whole lattice considering a linear coefficient of thermal expansion. The simulation assumed free moving atoms since calculations were made above melting temperature [35]. The simulation calculated the pair potential forces and in each calculation step rearranges the atom positions accordingly. After multiple steps, the position changes minimize and the final geometrical atom arrangement was found, which was used for the absorption calculation. As additional feature, the model includes Fermi band smoothening effects by considering the higher probability of electrons being in higher energetic states and the higher probability to activate lower-energy electrons at increasing temperature. Those effects increase the absorption volume of an atom and counteract the reduced absorption volume by the increased distance of the atoms due to thermal expansion.

The absorption was calculated assuming that the photon is absorbed with a certain probability when in the volume around an atom. The unphysical assumption of continuous absorption was used to estimate the maximum absorption depth to where the photon energy is statistically completely absorbed. The absorption was calculated using Drude's theory [26] and Fresnel equations at randomly polarized light. Incoming laser photons were angled in order to describe the angle-impact on the absorptance. The model was implemented in MATLAB 2019.

The suggested model is based on several assumptions:

- Pure material without alloying elements and contaminations were assumed for the calculations. This includes that the properties are not exactly the same as for the used steel in the experiments.
- A simplified pair potential model was used, which is commonly used to described atom interactions, but cannot fully describe the complex metal lattice. Therefore, Coulomb interactions were added to the model to account for the long-distance effects. However, a sensitivity analysis showed that the atom positioning is comparable after a minimum of three calculation steps and has a minor effect on the absorption calculation (∼1 % deviation).
- Due to the heavy calculation, a relatively small cube was selected in order to calculate multiple scenarios. However, atom positioning was seen to show comparable results at increased number of atoms.

The uncertainties of the calculated absorption values were estimated considering the inclination of the rays and the partial penetration of those due to the limited number of modelled atoms and the variations of the calculated values from the third to the seventh calculation step around the quasi-static state, which was in the range of 1 %.

## 3 Results and discussion

### 3.1 Absorption mechanisms

Light absorption is a photon-electron energy transfer, lifting electrons to higher energetic states. Two main mechanisms are known to occur in metals, namely the inter- and intraband absorption. The interband absorption lifts bound electrons to higher energy levels, while intraband absorption includes free electrons. Classical mechanics describing the complex permittivity as a function of free electron oscillations (intraband) and bound electron oscillations (interband) can be used [19]. Interband absorption leaves a hole in the valence band and adds electrons to the conduction band [19]. In the far-infrared wavelength range, photon energies are too small to initiate interband transitions and permittivity is defined only by intraband transitions [19], which was e.g. considered by Drude's theory.

It is known that metals show the so-called X-point, a wavelength, at which the temperature coefficient of absorptances changes its sign [36] due to the different temperature coefficients for interband and intraband transitions [37]. Free electrons show an increasing kinetic energy at higher temperatures, while phonon population grows, which increases the electron-phonon collision probability [27]. This leads to an increased intraband absorption at higher temperatures.

The more complex interband absorption depends on the band structure of the metal. In general, the broadening of absorption bands due to smoothening of the Fermi energy distribution function at increasing temperatures leads to a decreasing absorption [17]. The X-point for many metals is in the range of 1 μm wavelength. However, for transmission metals, like iron, the theory shows deviations, which indicates that interband transitions can contribute within the entire spectral range [38]. Since during the phase transition when melting, the lattice structure changes, and the number of conduction electrons increases with metal density and DC resistivity [39].

However, the temperature itself is not the only variable for describing absorption. During laser pulse absorption measurements, it was shown that the laser beam intensity (power per illuminated area) plays an important role [40], while the tendency was seen that at higher intensities, the intraband transition becomes more dominant [31]. The radical absorption increase was shown not to be a sole temperature effect, since also two-photon or even three-photon absorption can take place [41]. At very high intensities of pulsed laser beams, an absorption of 80 %–90 % was predicted by a relativistic analytical model [42]. The variation of the complex refractive index in depth of the material surface was assumed to impact absorption as well [31]. In addition, metal impurities in the crystal lattice can increase vibrations due to the increased temperature. Surface structures can also alter the total absorptance of a surface, which can be induced by surface roughness or even nano and micro surface structures from ablation [19].

**Fig. 2:**
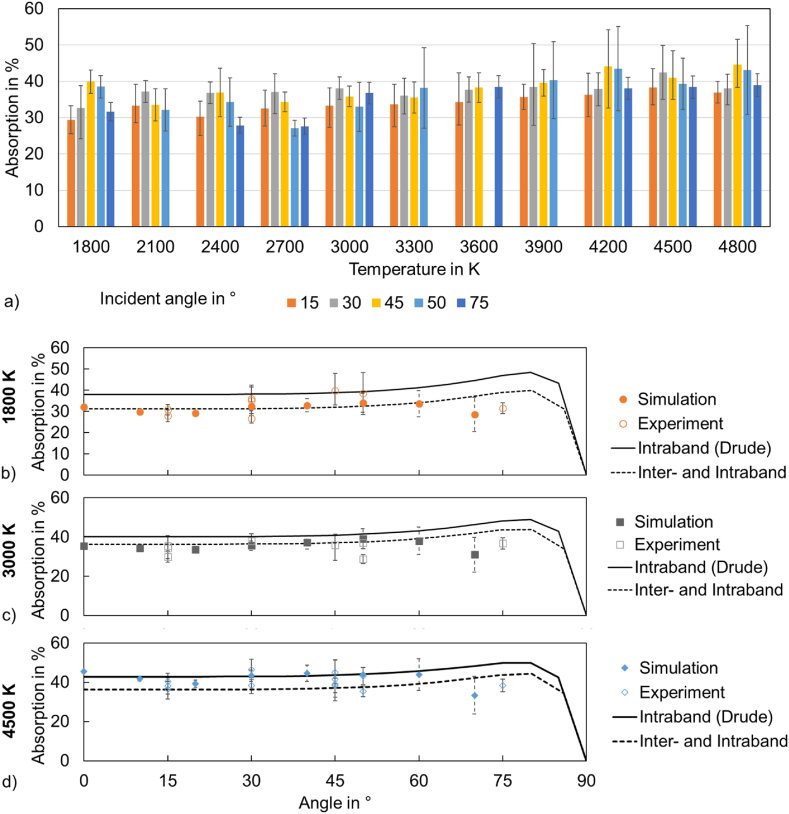
a) Experimental absorption measurements and b)-d) experimental and simulated absorption values at different temperatures compared with theoretical predictions.

In order to investigate the absorption mechanisms of metals at high temperatures, an adapted reflectometry measurement method and a molecular dynamic simulation were conducted in this work and the results were compared to theoretical predictions (Fig. 2).

A general trend to higher absorption at increasing temperatures can be seen in the experiments for all measured incident angles (Fig. 2a). At large angles, a slight absorption decrease can be seen just below the boiling temperature (∼3000 K). These trends are also seen in the simulation results (Fig. 2b). At small angles, the measured and simulated absorption values follow the theoretical curves. At lower temperatures the values are closer to the assumptions of inter- and intraband absorption and at 4500 K to the intraband absorption. At larger angles, the theory predicts the Brewster maximum of intraband and combined inter- and intraband calculations. However, the measured and simulated data show comparably low values of absorption maxima. This effect could be related to multi-interface absorption effects. Possible reasons can be phenomena occurring in the Knudsen layer or surface layering effects. Surface layering was seen to be able to induce a very low Brewster angle comparable to the measurements [43].

The measurement and simulation results show reasonable values and tendencies compared to literature data found in process simulations and comparable measurements at high temperatures. In process simulations, often absorption values must be assumed and estimated. Using a constant absorptivity of 0.35 in modelling 316L powders or 0.13 for flat S705 steel, good quantitative agreement was found with experiments [44,45]. However, Abadi et al. [1] found that the typically assumed absorption value of 0.34 for Ti-alloys underestimates the absorptance, which indicates that the determined absorption values of the current work show the right tendency to higher values at increasing temperatures. Implemented absorptivity models (e.g. by Ref. [45]) based on absorptivity models of Mahrle & Beyer [46] and Yang et al. [47] show a slight increase in absorption values of 316L stainless steel at increasing temperatures below boiling temperature comparable to the data of the current work.

### 3.2 Atomic positioning and fermi band broadening

Since only electrons near the Fermi-energy can be excited by incoming photons, typically, only intraband absorption is assumed in metals. However, the simulation and experimental results (Fig. 2b–d) show values that are closer to the theoretical absorption curve where intra- and interband absorption are considered. This indicates that interband absorption might play a larger role in metal absorption at high temperatures than thought. Possible explanations can be found in either increasing effects of Fermi band broadening of single atoms or geometrical effects of the bulk due to changed atomic bonding forces at elevated temperatures.

1) Fermi band broadening

It is known that interband absorption occurs when the photon energy is high enough, which is valid in the visible and ultraviolet spectrum for noble metals [27]. However, for transition metals, interband absorption can start at very low photon energy [27]. In those systems, the bands intersect the Fermi level and the Fermi curve significantly smoothens at higher temperatures [17]. The smoothening leads to a higher probability of electrons being present in conduction bands. This effect can explain the general tendency to higher absorption values at higher temperatures (Fig. 2a) due to intraband absorption.

Intraband absorption creates holes in the valence band. This means that arbitrary low energetic electrons can have the chance to be excited to higher energy levels by photon activation. Therefore, the virtual volume of the atom that can absorb photon energy should increase at higher temperatures and more electrons should be available for intraband as well as interband absorption. This can explain the tendency of absorption following the theoretical curve where interband absorption is considered (Fig. 2b–d).

2) Geometrical effects of atom arrangement

**Fig. 3:**
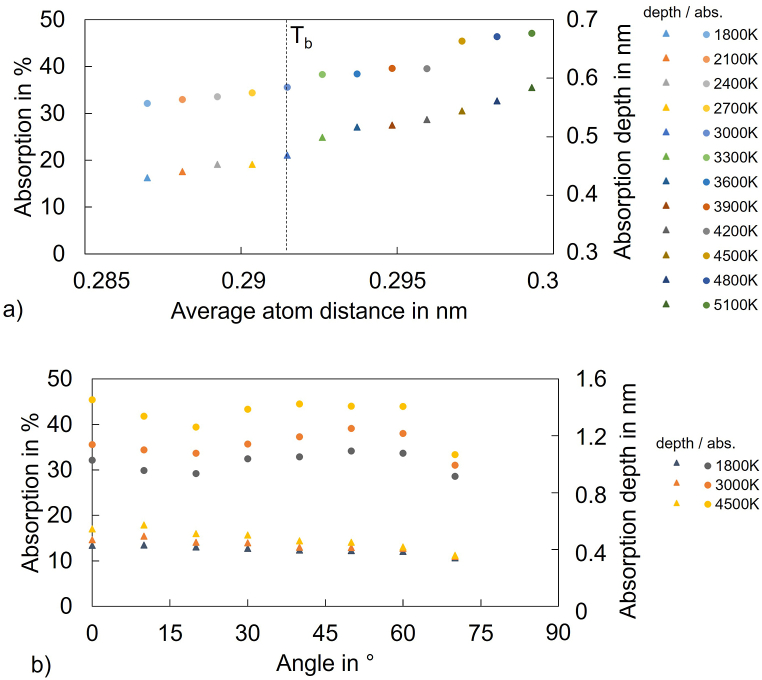
Absorption and absorption depth values from simulations at a) different temperatures (perpendicular laser incidence 0°) and b) different incident laser beam angles.

On the other hand, thermal expansion should lead to atom separation at increasing temperature, leading to less absorbing volume. However, the measurements (Fig. 2a) and the simulation results show that the absorption values and the absorption depths between melting and boiling temperature show a slight increase (Fig. 3a). Although the atom distance increases due to thermal expansion effects, the absorption seems to show only slight changes, which can be explained by the effect of increased absorptance due to increased interband absorption. In addition, it is known that the phonon population increases at increasing temperatures [26] leading to an increased electron-phonon energy transfer probability, which can support the absorptance.

**Fig. 4:**
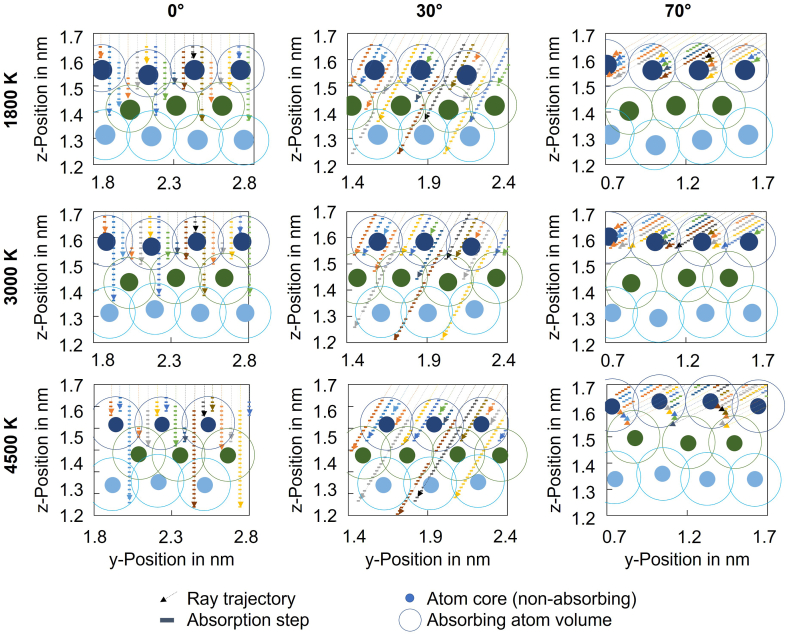
Sketch of surface atoms and laser rays being absorbed at different temperatures and incident laser beam angles.

Also the surface characteristics can impact the absorptance. Close to the melting temperature, surface roughening of the semi-solid material occurs, leading to an increased absorptance due to multiple reflections and absorption [19]. Those effects lead to an increased probability of photon absorption (Fig. 3, Fig. 4). Since such multiple reflection effects due to surface roughness were not considered in the simulation, the simulated absorption depth increase above boiling temperature must be related to the arrangement of the surface atoms that separate in combination with an increase of free electron density due to Fermi band broadening.

When melting metals, the solid-liquid phase change leads to an increase of conduction electrons, a higher density and an increase of the electrical resistivity due to the increased amount of lattice imperfections [39]. The increase of metal density and resistivity originates from the loss of long-range forces in the crystal order [42] and leads to a reduced number of direct neighbors of the atoms [35]. At further thermal expansion above the melting temperature, the resistance to expansion decreases. The trough of the Lennard-Jones potential is lower at higher temperatures [34]. Above boiling temperature, even a collapse of the system would be energetically possible. The ions even statistically gain more conduction electrons (Fermi band broadening) and must therefore show increased Coulomb retraction forces due to the statistically higher charge. This effect even leads to the increased separation of ions in the simulations (Fig. 3a). This is avoided due to the electron jellium gaining charge and holding the lattice together [48]. The bulk impact becomes therefore more important than the short-range atomic/ionic bonding forces.

Above boiling temperature, additional surface roughening occurs due to local ejections of atoms. This leads to multiple reflections on the surface and thereby to an increased absorption [19]. In addition, photons can penetrate deeper below the original material surface since vaporized atoms provide additional space. The loss of available conduction electrons in the surface due to atom ejection can explain that the intraband absorption is not dominating even at 4500 K. On the other hand, the atoms denote a loss of phonons that can interact with the remaining excited electrons, which makes the energy transfer more difficult.

The angle-dependent measurements and the simulation results (Fig. 2b–d) show a general tendency to higher absorption at angles around 30°–45° and a decreased absorption at higher angles. The general tendency of having an absorption peak is known and called the Brewster angle, where p-polarized light is almost completely absorbed. This Brewster angle is usually found at high angles (near 90°) [27]. The results of this work, however, indicate that the Brewster angle is decreased at such high temperatures.

## 4 Conclusions

Absorption values above melting temperature of metals are still often unknown and are mainly based on theoretical assumptions or reflect only the total absorptance of a complex system. Therefore, measurements in combination with a simulation model were used to derive absorption data at high temperatures and derive explanations for the observed characteristics. In addition to the intraband absorption of conduction band electrons (Drude), interband absorption seems to contribute to the total absorption even at high temperatures. It was shown that the balance between geometrical atom arrangement effects and the Fermi band broadening can be the reason for the observed and calculated increased absorption values at temperatures above boiling temperature and the decrease of the Brewster angle.

## Data availability statement

Data will be made available on request.

## CRediT authorship contribution statement

**Joerg Volpp:** Conceptualization, Data curation, Formal analysis, Funding acquisition, Investigation, Methodology, Project administration, Resources, Software, Validation, Visualization, Writing – original draft, Writing – review & editing.

## Declaration of competing interest

The authors declare the following financial interests/personal relationships which may be considered as potential competing interests: Joerg Volpp reports financial support was provided by 10.13039/501100004359Swedish Research Council. Joerg Volpp reports a relationship with Lulea University of Technology that includes: employment.
